# Electrocardiogram: his bundle potentials can be recorded noninvasively beat by beat on surface electrocardiogram

**DOI:** 10.1186/s12872-017-0516-3

**Published:** 2017-03-15

**Authors:** Gaopin Wang, Renguang Liu, Qinghua Chang, Zhaolong Xu, Yingjie Zhang, Dianzhu Pan

**Affiliations:** 1 The Cardiovascular Institute of the First Affiliated Hospital of Liaoning Medical University, Renmin Street, Jinzhou, 121000 Liaoning Province China; 2 Department of Respiration Medicine, The First Affiliated Hospital of Liaoning Medical University, Renmin Street, Jinzhou, 121000 Liaoning Province China

**Keywords:** Electrocardiogram, His bundle electrogram, New wavelets, His bundle potential

## Abstract

**Background:**

The micro waveform of His bundle potential can’t be recorded beat-to-beat on surface electrocardiogram yet. We have found that the micro-wavelets before QRS complex may be related to atrioventricular conduction system potentials. This study is to explore the possibility of His bundle potential can be noninvasively recorded on surface electrocardiogram.

**Methods:**

We randomized 65 patients undergoing radiofrequency catheter ablation of paroxysmal superventricular tachycardia (exclude overt Wolff-Parkinson-White syndrome) to receive “conventional electrocardiogram” and “new electrocardiogram” before the procedure. His bundle electrogram was collected during the procedure. Comparative analysis of PA_s_ (PA interval recorded on surface electrocardiogram), AH_s_ (AH interval recorded on surface electrocardiogram) and HV_s_ (HV interval recorded on surface electrocardiogram) interval recorded on surface “new electrocardiogram” and PA, AH, HV interval recorded on His bundle electrogram was investigated.

**Results:**

There was no difference (*P* > 0.05) between groups in HV_s_ interval (49.63 ± 6.19 ms) and HV interval (49.35 ± 6.49 ms). Results of correlational analysis found that HV_S_ interval was significantly positively associated with HV interval (*r* = 0.929; *P* < 0.01).

**Conclusions:**

His bundle potentials can be noninvasively recorded on surface electrocardiogram. Noninvasive His bundle potential tracing might represent a new method for locating the site of atrioventricular block and identifying the origin of a wide QRS complex.

## Background

An electrocardiogram exam plays an irreplaceable role in the diagnosis of arrhythmia [1]. To our knowledge, the potentials of specialized conduction system on surface electrocardiogram have not been recorded yet in human. Therefore, it is difficult to evaluate the sinoatrial function, locate the site of atrioventricular block and identify the origin of a wide QRS complex on surface. Although sinoatrial node potential [2–5] and His bundle potential [6–15] can be recorded in intracardiac electrophysiology study, it isn’t in a wide range of applications due to its invasive exam. With the clinical expectation, we used new electrocardiogram machine (model PHS-A10) and not only the P-QRS-T waves but also the micro-wavelets on surface electrocardiogram were firstly recorded in healthy and arrhythmia volunteers. In healthy and arrhythmias volunteers, we have found that the micro-wavelets before QRS complex (overlapped on P wave and in PR segment) may be related to atrioventricular conduction system potentials [16, 17]. In this study, we further verified the relationships between the micro-wavelets before QRS complex and atrioventricular conduction system potentials. According to the position and characteristics of these micro-wavelets before QRS complex, PR interval recorded on “new electrocardiogram” was divided into three intervals: PA_s_ (PA interval recorded on surface electrocardiogram) interval, AH_s_ (AH interval recorded on surface electrocardiogram) interval and HV_s_ (HV interval recorded on surface electrocardiogram) interval. Comparative analysis of PA_s_, AH_s_ and HV_s_ interval recorded on surface “new electrocardiogram” and PA, AH, HV interval recorded on His bundle electrogram was investigated in 65 patients undergoing radiofrequency catheter ablation of paroxysmal superventricular tachycardia. Now we present our preliminary analysis.

## Methods

### Materials

Sixty-five patients who were proved to have a paroxysmal supraventricular tachycardia formed the subjects of this study in the First Affiliated Hospital of Liaoning Medical University. There were 31 males and 34 females, with a mean age of 52 ± 13 years (ranging from 14 to 84 years). All patients were included 4 groups according to the intracardiac electrophysiology study: 33 patients with atrioventricular nodal reentrant tachycardia; 29 patients with atrioventricular reentrant tachycardia (exclude overt Wolff-Parkinson-White syndrome); one patient with atrial tachycardia; two patients with paroxysmal atrial fibrillation. We used a “new electrocardiogram” machine (model PHS-A10) designed and developed by EmCG US company to record surface electrocardiogram. The intracardiac electrophysiology study was performed by an electrophysiological recording and the analysis system Model CardioLab 7000 (GE Co., USA).

### Methods

Resting supine 12-lead electrocardiograms in sinus rhythm and His bundle electrogram in sinus rhythm were recorded before the intracardiac electrophysiology study. Two independent observers who knew the purpose of the study but were blinded to the study design and the results of all investigations evaluated the electrocardiograms and His bundle electrogram.

1. Recording conditions: The subject’s skin was firstly pretreated with sandpaper, and then special electrodes (non-cytotoxic silver/silver chloride substrate) are used to perform the test, in which the arrangement of electrodes were the same as that in a 12 lead electrocardiogram. In addition, both “new electrocardiogram” [It was coined the term of saahECG (SAN-Atrial-AVN-His ECG) by the developers] and “conventional electrocardiogram” have been detected synchronously by the “new electrocardiogram” machine. Moreover, the scanning speed (25 mm/s) and amplitude (10 mm/mV), and the measuring speed (25 mm/s) and amplitude (20 mm/mV) in long trace leads were selected. The micro-wavelets before P wave, before QRS complex (in P wave and PR segment) and after QRS complex (ST segment and upstroke of T wave) can be recorded by the “new electrocardiogram” machine (Fig. 1a, b and c segments, the wavelet mechanism refers to discussion part 1).

**Fig. 1:**
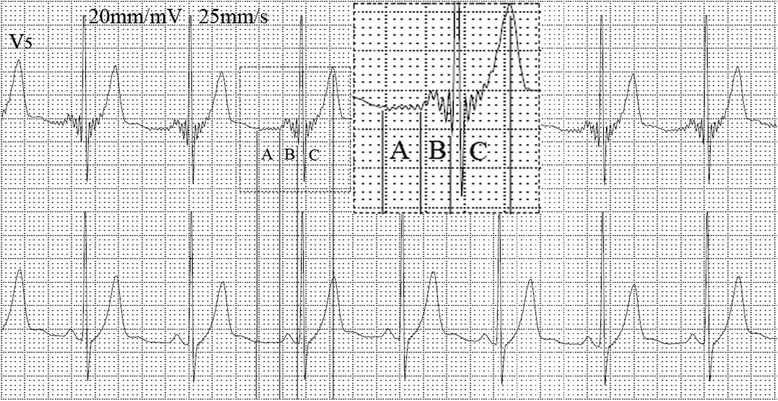
The “new electrocardiogram” and “conventional electrocardiogram” are recorded simultaneously. The upper lead is “new electrocardiogram” and the lower is “conventional electrocardiogram” in lead V_5_. New wavelets are clearly recorded before P wave (**a**), before QRS complex (**b**) and after QRS complex (**c**)
2. Mapping of HV_s_, AH_s_ and PA_s_ intervals on surface electrocardiogram was as following: PA_s_ interval (the time interval from the initiation of the P wave to the first notch of the P wave), AH_s_ interval (the time interval from the first notch of the P wave to the initiation of the second wavelet with higher amplitude closed to the QRS complex) and HV_s_ interval (the time interval from the initiation of the second wavelet with higher amplitude closed to the QRS complex to the start of the QRS complex). The values of each intervals measured by the computer were seen in Fig. 2 (principle refer to discussion part 2). These micro-wavelets (the paper speed of electrocardiogram was 25 mm/s and the gain was 20 mm/mV) were amplified (four magnification) on the computer to determine the distinct measuring point (always in leads II or V_5_). The values of each intervals (ms) measured by the computer.

**Fig. 2:**
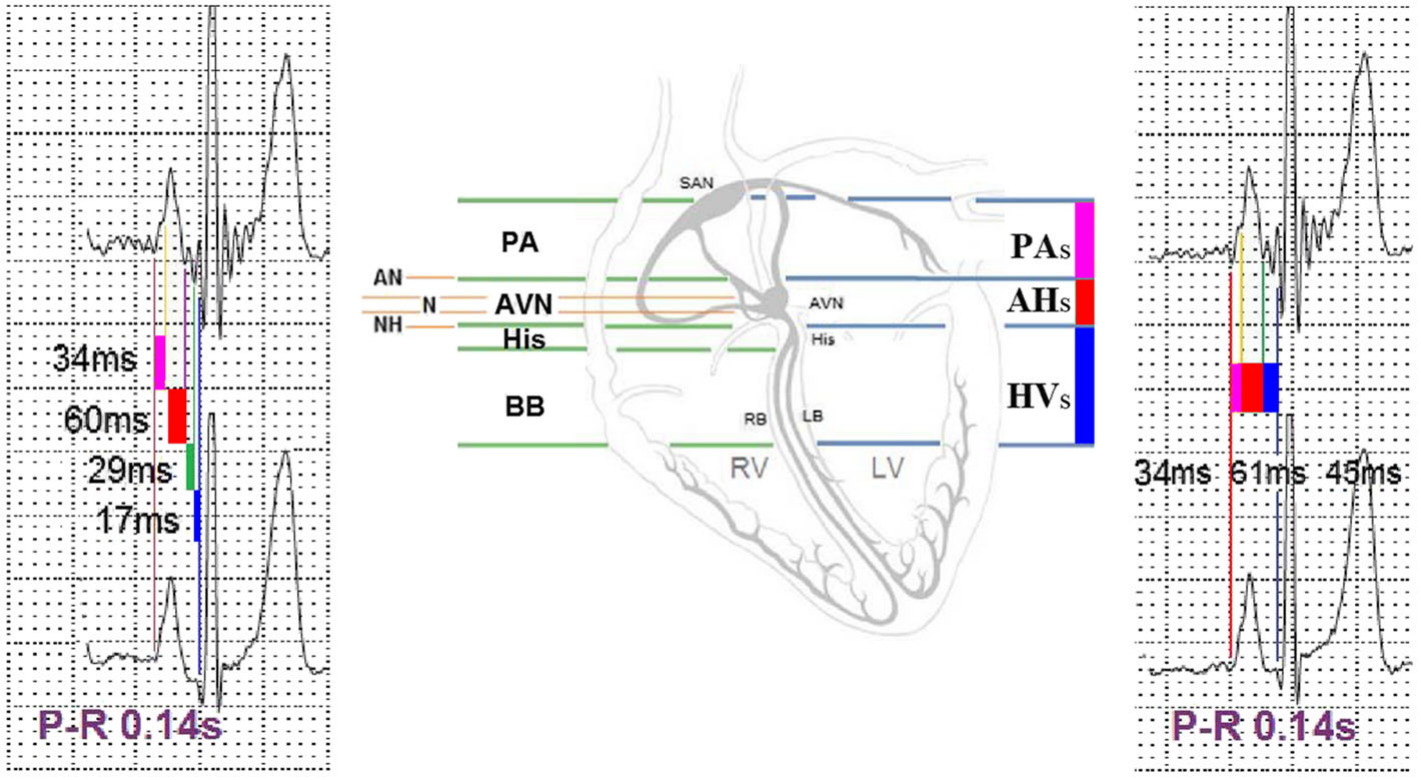
According to the micro-wavelets before QRS complex, PR interval is the summation of three intervals: PA_s_ interval (the time interval from the initiation of the P wave to the first notch of the P wave), AH_s_ interval (the time interval from the first notch of the P wave to the initiation of the second wavelet closed to the QRS complex) and HV_s_ interval (the time interval from the initiation of the second wavelet closed to the QRS complex to the start of the QRS complex). PA_s_ indicates that PA interval recorded on surface electrocardiogram; AH_s_ indicates that AH interval recorded on surface electrocardiogram; HV_s_ indicates that HV interval recorded on surface electrocardiogram
3. Details concerning electrophysiologic study (including His bundle electrogram record and PA, AH, HV interval measurement) were obtained as previously described [18]. They were recorded at paper speeds of 100 mm/sec and be analyzed by ruler in computers (manual selection of measurement points).

### Statistical analysis

The results were given as means ± s.d., Paired t tests were used to compare the index of “new electrocardiogram” and His bundle electrogram. The correlation between the same variables measured by the two methods was analyzed by using Pearson correlation analysis. All statistical analysis was performed using SPSS 16.0. A *P*-value <0.05 was considered significant.

## Results

1. Comparative analysis of intervals recorded on “new electrocardiogram” and His bundle electrogram

**Fig. 3:**
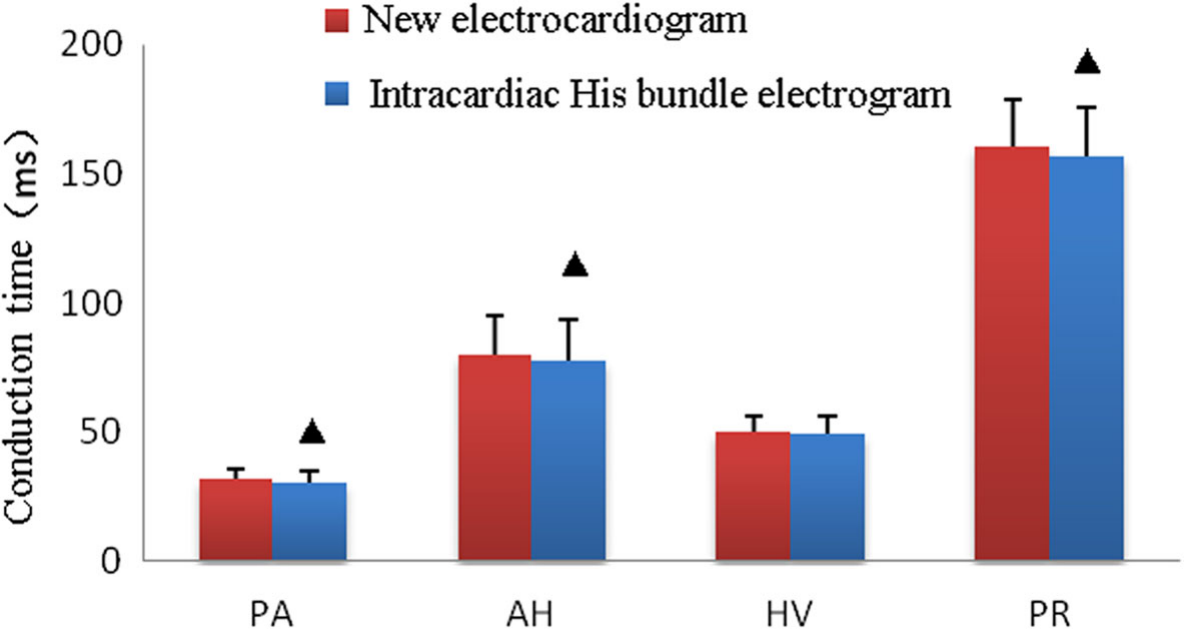
Comparative study of PA_s_, AH_s_ and HV_s_ interval recorded on “New electrocardiogram” and PA, AH and HV interval recorded on His bundle electrogram. Compared with intracardiac electrophysiology study, ▲ indicates *P* < 0.05

**Fig. 4:**
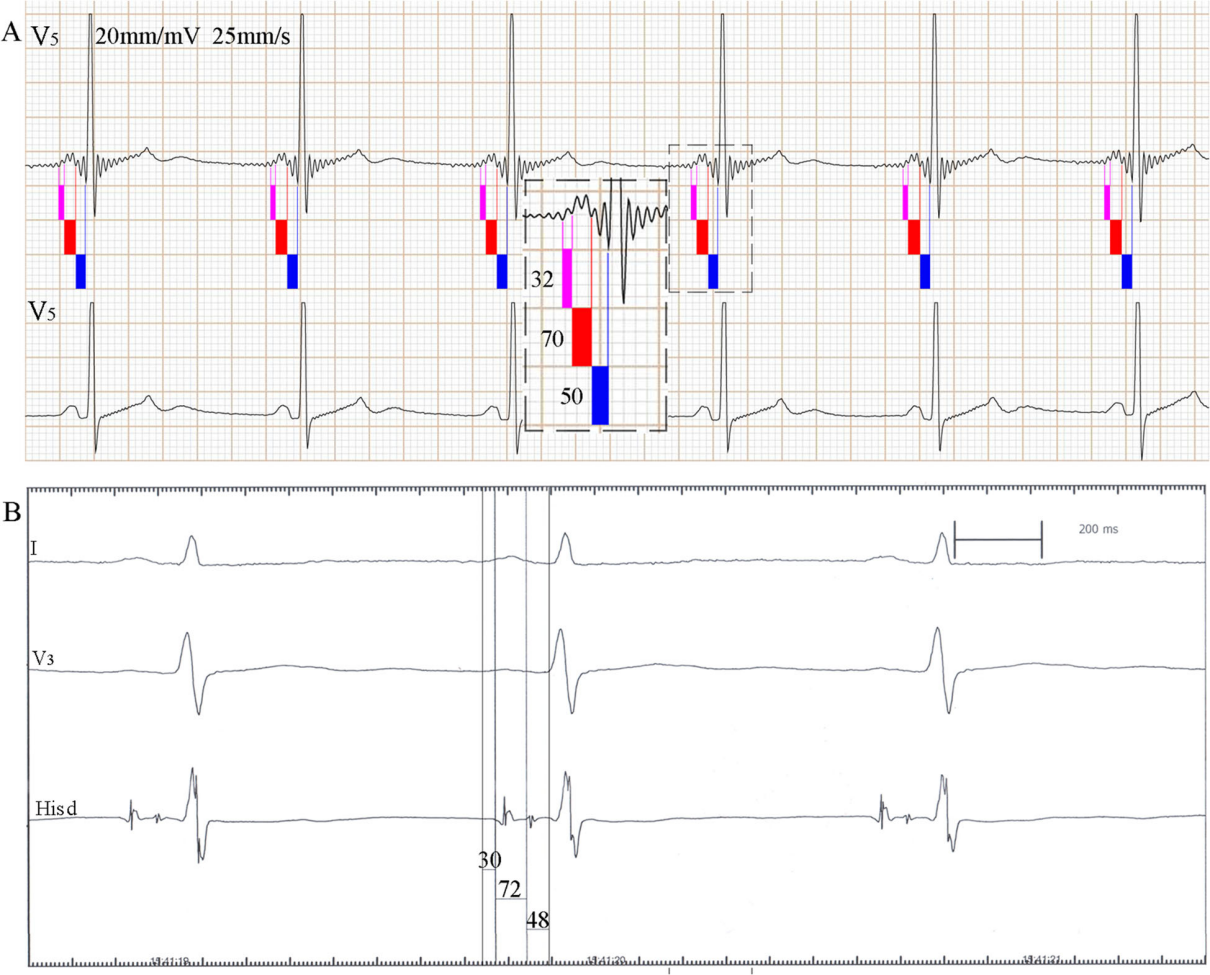
Comparative study of PA_s_, AH_s_ and HV_s_ interval recorded on “New electrocardiogram” and PA, AH and HV interval recorded on His bundle electrogram in one subject. **a** shows the new electrocardiogram (*upper trace*) and conventional electrocardiogram (*lower trace*). **b** shows the His bundle electrogram. The PA_s_, AH_s_ and HV_s_ intervals on new electrocardiogram are consistent with the intra-atrial measurement (PA, AH and HV interval). Figure is representative of one subject with similar results

There were no difference (*P* > 0.05) between groups in HV_s_ interval recorded on “new electrocardiogram” and HV interval recorded on His bundle electrogram. There were differences (*P* < 0.05) between groups in PA_s_, AH_s_, PR interval recorded on “new electrocardiogram” and PA, AH, PR interval recorded on His bundle electrogram, as shown in Table 1, Figs. 3 and 4.

**Table 1:** Comparative analysis of intervals recorded on “new electrocardiogram” and His bundle electrogram

Group	PA (ms)	AH (ms)	HV (ms)	PR (ms)
New electrocardiogram	31.34 ± 4.17 ^a^	79.86 ± 15.35 ^a^	49.63 ± 6.19	160.83 ± 17.92 ^a^
His bundle electrogram	30.14 ± 4.91	77.20 ± 16.04	49.35 ± 6.49	156.52 ± 19.09
t	3.269	2.113	0.927	3.288
P	0.002	0.038	0.357	0.002

Note: Values are mean means ± s.d. *n* = 65. Compared with His bundle electrogram, ^a^ indicate *P* < 0.05

2. Pearson correlation analysis between PA_s_, AH_s_, HV_s_, PR_s_ intervals recorded on “new electrocardiogram” and PA, AH, HV, PR interval recorded on His bundle electrogram

**Fig. 5:**
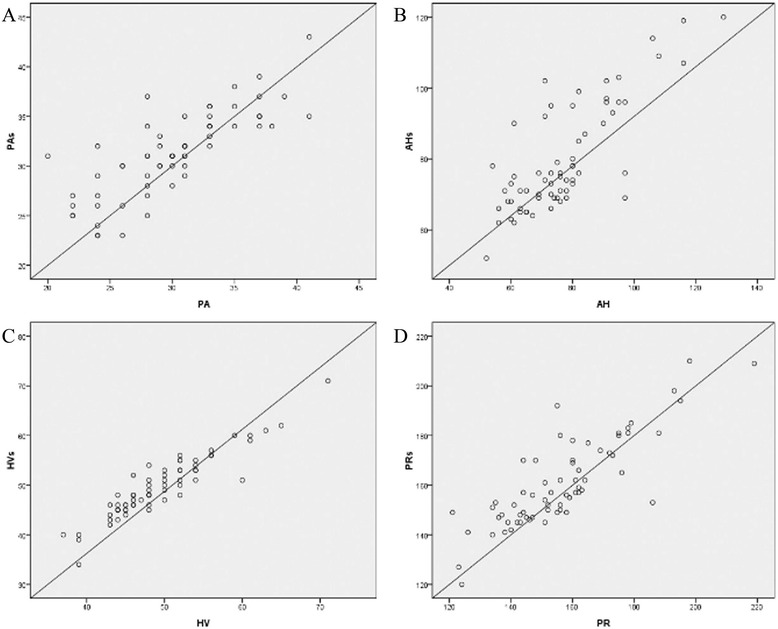
Pearson correlation analysis between PA_s_, AH_s_, HV_s_, PR_s_ interval recorded on “new electrocardiogram” and PA, AH, HV, PR interval recorded on His bundle electrogram. **a** shows Pearson correlation analysis between PA_s_ interval and PA interval; **b** shows Pearson correlation analysis between AH_s_ interval and AH interval; **c** shows Pearson correlation analysis between HV_s_ interval and HV interval; **d** shows Pearson correlation analysis between PR_s_ interval and PR interval; PA_s_ was significantly positively associated with PA interval (*r* = 0.800; *P* < 0.01); AH_s_ interval was significantly positively associated with AH interval (*r* = 0.792; *P* < 0.01); HV_s_ interval was significantly positively associated with HV interval (*r* = 0.929; *P* < 0.01); PR_s_ interval was significantly positively associated with PR interval (*r* = 0.839; *P* < 0.001)

Results of correlational analysis found that PA_s_ was significantly positively associated with PA interval (*r* = 0.800; *P* < 0.01); AH_s_ interval was significantly positively associated with AH interval (*r* = 0.792; *P* < 0.01); HV_s_ interval was significantly positively associated with HV interval (*r* = 0.929; *P* < 0.01); PR_s_ interval was significantly positively associated with PR interval (*r* = 0.839; *P* < 0.001), as shown in Fig. 5.

## Discussion

1. Why “New electrocardiogram” can record the micro waveform before QRS complex which conventional electrocardiogram couldn’t?

The PHS-A10 electrocardiogram is a new device created by EmCG US company using the latest technology of the software and the hardware, and the signal processing technology. These are based on a collection of various US patents and International patents. The PhySio’s PHS-A10 is highlighted by its success in employing a novel Adaptive Mixture Technology within the electrocardiogram Signal Spectrum enabled by PhySio’s Smart Data Acquisition Module. Along with traditional electrocardiogram scanning/recording, PHS-A10 is able to accurately extract a variety of time-domain electrocardiogram electric potentials in the 0-150Hz range, and perform automated integrating signals recognition. Therefore, not only the P-QRS-T waves but also the micro-wavelets before P wave, before QRS complex (overlapped on P wave and in PR segment), after QRS complex (ST segment and upstroke of T wave) can be recorded on surface electrocardiogram (Fig. 1a, b, c segments). Besides, on base of new wavelets recorded by “new electrocardiogram”, the Electrophysiocardiogram has further been created by EmCG US company. Electrophysiocardiogram has separated the acquired convoluted signals into a plurality of different linear wave forms in various frequency ranges, for examples, for the frequency range from 1 to 150 Hz (conventional electrocardiogram), and it has been divided into 32 linear wave forms. Therefore, these wavelets can be revealed more clearly [19]. The resulting electrocardiogram wavelet signals are displaying the natural signals of various parts of the heart (before P wave, before QRS complex and after QRS complex) without interfering artifacts in our 100 healthy volunteers [17]. In our study, we only discuss the wavelet before QRS complex.

2. According to the micro-wavelets before QRS complex, PR interval was divided into PA_s_, AH_s_ and HV_s_ interval on “New electrocardiogram”

(1) In 100 healthy individuals, we have found that the new electrocardiogram can record characteristic wavelets before QRS complex: two wavelets in PR segment with higher amplitude closest to QRS complex and one to three wavelets with lower amplitude before these two wavelets) [17]. The wavelets in P wave and PR segment might be related to atrioventricular node and His bundle- bundle potentials due to cardiac anatomy and the sequence of electrical activation. (2) In patients with atrial (atrial contraction, atrial tachycardia, atrial flutter, atrial fibrillation) and junctional arrhythmias (premature junctional contraction, junctional escape), we also have found the characteristic wavelets above. In patients with ventricular arrhythmias (premature ventricular contraction, ventricular tachycardia, ventricular pacing), there was no micro waveform before the wide QRS complex [17]. These suggested the wavelets before QRS complex might be related to atrioventricular conduction system potentials. (3) In patients with second degree atrioventricular block type I (atrioventricular node level block), we have found that two wavelets in PR segment with higher amplitude closest to QRS complex were constant and several wavelets with lower amplitude before these two wavelets (overlapped in P wave and after the P wave) were progressive increased according to the progressive lengthening PR interval [16]. These suggested this two wavelets closed to the QRS complex with high amplitude might be related to His bundle and bundle branch potentials. (4) Based on the above study, we preliminary recognized that PR interval was divided into PA_s_ interval (the time interval from the initiation of the P wave to the first notch of the P wave), AH_s_ interval (the time interval from the first notch of the P wave to the initiation of the second wavelet with higher amplitude closed to the QRS complex) and HV_s_ interval (the time interval from the initiation of the second wavelet with higher amplitude closed to the QRS complex to the start of the QRS complex). A finding in our 100 healthy individuals was that the PA_s_, AH_s_ and AH_s_ intervals were consistent with the intra-atrial measurement PA, AH and HV intervals [18]. In the patient with first-degree atrioventricular block at atrioventricular nodal level which verified by intracardiac electrogram, we have observed on new electrocardiogram that two wavelets in PR segment with higher amplitude closest to QRS complex maintained unchanged and the number of wavelets with lower amplitude before these two wavelets was increased. Thus, these confirmed that PR interval was divided into PA_s_, AH_s_ and HV_s_ interval.

3. Comparative study of PA_s_, AH_s_ and HV_s_ interval recorded on “New electrocardiogram” and PA, AH and HV interval recorded on His bundle electrogram

(1) A finding of Paired t tests in our study in 65 paroxysmal supraventricular tachycardia underwent “New electrocardiogram” and His bundle electrogram (these two weren’t simultaneously recorded) was that there was no difference (*P* > 0.05) between groups in HV_s_ interval and HV interval. Results of correlational analysis found that HV_S_ interval was significantly positively associated with HV interval (*r* = 0.929; *P* < 0.01). These suggested two wavelets closed to the QRS complex with high amplitude in HV_s_ interval was His bundle and bundle branch potentials on surface electrocardiogram. (2) A finding of Paired t tests revealed there were differences (*P* < 0.05) between groups in AH_s_ interval (79.86 ± 15.35 ms) and AH interval (77.20 ± 16.04 ms). The means difference of AH_s_ and AH interval was in a few milliseconds and was in the range of physiological changes (autonomic nerve change) of AH interval (20–50 ms) [5]. Therefore, there was no clinical significance. Besides, results of correlational analysis found that AH_s_ interval was significantly positively associated with AH interval (*r* = 0.792; *P* < 0.01). Therefore, the wavelets (overlapped in P wave and after the P wave) in AH_s_ interval may be atrioventricular nodal potential.

### Study limitations

(1) “New electrocardiogram” and His bundle electrogram weren’t simultaneously recorded. Therefore, autonomic nerve change may have effect on atrioventricular nodal, leading to differences between groups in AH interval and PR interval. (2) The paper speed of “New electrocardiogram” weren’t consistent with His bundle electrogram. The paper speed of “New electrocardiogram” was 25 mm/s and the paper speed of His bundle electrogram was 100 mm/s. If the paper speed of “New electrocardiogram” was 100 mm/s, the acute angle of the wavelet before QRS complex became blunt. Therefore, it was difficult to determine the starting point. To reduce the impact of paper speed, the body surface measurements were amplified (four magnification) on the computer. It can not completely exclude the impact of paper speed. (3) In AH_s_ interval, the A point was the initiation of the first notch of the P wave (not an acute angle), but the starting point of A wave in His bundle electrogram was clear. Although these limitations didn’t have effect on clinical significance of our result, the normal scope of PA_s_, AH_s_ and HV_s_ interval can’t be applied mechanically according to the intracardiac electrophysiology study. It still required statistical analysis of large sample.

## Conclusions

In conclusion, our study revealed that His bundle potential can be noninvasively recorded beat by beat in human. It has only begun with a prologue for a new research and still requires a great number of clinical and animal experiments in order to verify the clinical significance, normal scope, characteristics and mechanism of these micro waveforms. The purpose of this study is to put forth in the hope that more doctors and scientists will join hand to greet the second spring of the electrocardiogram.

## Abbreviations

AH_s_: AH interval recorded on surface electrocardiogram
HV_s_: HV interval recorded on surface electrocardiogram
PA_s_: PA interval recorded on surface electrocardiogram
